# In vitro cytocompatibility and antibacterial studies on biodegradable Zn alloys supplemented by a critical assessment of direct contact cytotoxicity assay

**DOI:** 10.1002/jbm.b.35147

**Published:** 2022-08-26

**Authors:** Maria Wątroba, Wiktor Bednarczyk, Piotr K. Szewczyk, Jakub Kawałko, Krzysztof Mech, Alina Grünewald, Irem Unalan, Nicola Taccardi, Gabriela Boelter, Manuel Banzhaf, Caroline Hain, Piotr Bała, Aldo R. Boccaccini

**Affiliations:** ^1^ Laboratory for Mechanics of Materials and Nanostructures Empa, Swiss Federal Laboratories for Materials Science and Technology Thun Switzerland; ^2^ Faculty of Metals Engineering and Industrial Computer Science AGH University of Science and Technology Krakow Poland; ^3^ Faculty of Materials Science and Engineering Warsaw University of Technology Warsaw Poland; ^4^ Academic Centre for Materials and Nanotechnology AGH University of Science and Technology Krakow Poland; ^5^ Department of Materials Science and Engineering, Institute of Biomaterials University of Erlangen‐Nuremberg Erlangen Germany; ^6^ Institute of Chemical Reaction Engineering University of Erlangen‐Nuremberg Erlangen Germany; ^7^ Institute of Microbiology and Infection and School of Biosciences University of Birmingham Birmingham UK; ^8^ Institute for Applied Laser Photonics and Surface Technologies ALPS Bern University of Applied Sciences Biel/Bienne Switzerland

**Keywords:** Zn alloys, biodegradation, cytotoxicity, tetrazolium salt‐based assay, antibacterial properties

## Abstract

In vitro cytotoxicity assessment is indispensable in developing new biodegradable implant materials. Zn, which demonstrates an ideal corrosion rate between Mg‐ and Fe‐based alloys, has been reported to have excellent in vivo biocompatibility*.* Therefore, modifications aimed at improving Zn's mechanical properties should not degrade its biological response. As sufficient strength, ductility and corrosion behavior required of load‐bearing implants has been obtained in plastically deformed Zn‐3Ag‐0.5Mg, the effect of simultaneous Ag and Mg additions on in vitro cytocompatibility and antibacterial properties was studied, in relation to Zn and Zn‐3Ag. Direct cell culture on samples and indirect extract‐based tests showed almost no significant differences between the tested Zn‐based materials. The diluted extracts of Zn, Zn‐3Ag, and Zn‐3Ag‐0.5Mg showed no cytotoxicity toward MG‐63 cells at a concentration of ≤12.5%. The cytotoxic effect was observed only at high Zn^2+^ ion concentrations and when in direct contact with metallic samples. The highest LD_50_ (lethal dose killing 50% of cells) of 13.4 mg/L of Zn^2+^ ions were determined for the Zn‐3Ag‐0.5Mg. Similar antibacterial activity against *Escherichia coli* and *Staphylococcus aureus* was observed for Zn and Zn alloys, so the effect is attributed mainly to the released Zn^2+^ ions exhibiting bactericidal properties. Most importantly, our experiments indicated the limitations of water‐soluble tetrazolium salt‐based cytotoxicity assays for direct tests on Zn‐based materials. The discrepancies between the WST‐8 assay and SEM observations are attributed to the interference of Zn^2+^ ions with tetrazolium salt, therefore favoring its transformation into formazan, giving false cell viability quantitative results.

## 1 INTRODUCTION

The growing demand for short‐term implants has resulted in the continuous development of biodegradable metallic materials to replace already used permanent implants made of stainless steel, cobalt‐ and titanium‐based alloys.^1^ Ease of manufacturing, optimal degradation rate, and biocompatibility make zinc (Zn) a competitive alternative to magnesium (Mg)‐ and iron (Fe)‐based alloys considered for biomedical applications, such as stents, fracture fixations, or sutures. Zn is an essential trace element in the human body as it participates in almost all metabolic reactions, for example, for numerous enzymes' proper activity, maintaining immune functions, and supporting protein and DNA syntheses.^2^ Additionally, Zn plays an essential role in cell division, cell growth, cell apoptosis regulation, and wound healing.^3^ The upper intake limit for Zn is estimated to be 15–40 mg per day.^4^ Zn deficiency affects nearly all physiological functions leading to a greater risk of infection, retardation or cessation of growth, bleeding tendency, hair loss, dermatitis, neuropathy, hypotension, or defects resulting in impaired parturition.^5^ However, an excess of Zn might lead to neurotoxicity problems and may damage vital organs, such as the kidney, liver, spleen, brain, and heart.^6^, ^7^ Although some in vitro biocompatibility research report cytotoxicity of Zn‐based materials,^8^, ^9^ in vivo feasibility studies have validated the biocompatibility of pure Zn and Zn alloys.^10^ Furthermore, since the first successful in vivo studies of implanting a pure Zn wire into rat aortae, Zn's outstanding physiological corrosion behavior and biocompatibility have been highlighted.^11^ The released Zn^2+^ ions or Zn‐based corrosion products can be absorbed by the surrounding tissues and excreted through the gastrointestinal route or urinated after being filtered through the kidneys.^5^, ^12^ Therefore, Zn^2+^ release during in vivo degradation is assumed to be safe and Zn is not considered to be toxic.

Nevertheless, as‐cast pure Zn's poor mechanical strength, brittleness, and recrystallization at room temperature (RT) pose significant challenges for load‐bearing applications, with their modification without altering their biodegradation behavior and biological properties problematic. Recently published results indicate that alloying with biocompatible elements, using different fabrication techniques and plastic deformation processes, or heat treatment can successfully address these fundamental issues.^10^, ^13^, ^14^ In particular, our previous research indicate that the mechanical properties of the Zn‐3Ag‐0.5Mg alloy fulfill the requirements for biodegradable implant materials.^15^, ^16^ The best strength/ductility combination obtained in the cold‐rolled Zn‐3Ag‐0.5Mg alloy reached 432 MPa of the ultimate tensile strength (UTS), 385 MPa of the yield strength (YS), and 34% of the total elongation to fracture (ε_F_) after short‐term heat treatment.^16^ These mechanical properties are significantly higher than those required for implant applications: ε_F_ > 15%, UTS > 300 MPa, and YS > 200 MPa for stents or YS > 230 MPa for bone fracture fixations.^11^ Recent studies indicate that Zn‐3Ag‐0.5Mg alloy is a promising material also in terms of corrosion properties.^17^ Evenly distributed precipitates of second phases form micro‐galvanic cells in the fine‐grained microstructure (high grain boundary density), which led to uniform corrosion without substantial localized corrosion. The formation of tiny pits on the entire samples' surface resulted in a corrosion rate of 21.8 ± 0.4 μm/year after 6‐month in vitro degradation studies performed in Hanks' solution. The value was higher compared to the degradation of pure Zn at 12.8 ± 0.2 μm/year. Similar studies are carried out for other biodegradable materials. In general, it was observed that alloying accelerates the corrosion compared to pure Zn, however, the values noted for Zn alloys remain in the same order of magnitude, when tested in the same salt‐based environment.^18^ When compared to other biodegradable metals, the corrosion rate of Zn‐based materials is placed between that of Mg and Fe. Mg‐based materials typically exhibit one to two orders of magnitude faster corrosion rate. Additionally, the process is accompanied by hydrogen gas evolution,^19^ undesirable for internal biomedical applications. In contrast, Fe is characterized by a significantly lower degradation rate than Zn, however, corrosion usually occurs locally, with the degradation products not being absorbed by the human body at an appropriate rate.^20^, ^21^

Silver (Ag) and Mg, as alloying additions in Zn, can be beneficial not only for mechanical strength and ductility enhancement, but also have a positive impact on the biological properties.^22^, ^23^ It has been reported that Mg^2+^ ions could positively impact MG‐63 cell viability, proliferation, and adhesion when tested under in vitro conditions. Mg is an essential nutrient for the human body, with the recommended daily allowance between 320 and 400 mg.^24^ It promotes protein synthesis and acts as an activator of many enzymes. In addition, Mg^2+^ supports essential biological processes, including bone formation, by interacting with osteoblastic cell integrins responsible for cell adhesion and stability.^25^, ^26^ Silver's most significant biological advantage is to enhance the antibacterial activity of Zn alloys.^27^, ^28^ The human tolerance level for Ag ranges from 0.4 to 27 μg/day.^29^

With the Zn‐3Ag‐0.5Mg alloy being a potential candidate for biodegradable implant applications, it is in vitro cytotoxicity testing against MG‐63 osteoblast‐like cells and antibacterial behavior evaluation against *Escherichia coli* (*E. coli*) and *S. aureus* (*Staphylococcus aureus*) was of high importance within the scope of this study. Additionally, pure Zn and the Zn‐3Ag alloy were also tested to serve as reference samples. For cytocompatibility tests, two common tetrazolium salt‐based cytotoxicity assays were used. Direct contact tests aimed at investigating the cellular response to Zn‐based material surfaces, while indirect tests were used to observe for signs of toxicity in response to differently‐concentrated fluid extracts of Zn‐based and control materials. These results were additionally validated via microscopic observations. Furthermore, the materials' antibacterial activity was evaluated by utilizing turbidity tests and placing the Zn‐based samples on Agar plates containing bacteria.

During in vitro cytotoxicity testing of the selected Zn‐based materials, it was found that the water‐soluble tetrazolium salt‐based (WST) colorimetric assay delivers false‐positive results on cell viability due to the interference of Zn^2+^ ions with tetrazolium salt included in the WST‐8 assay reagent. This discovery makes the present work particularly appealing for researchers working in the field of general biological characterization of newly designed Zn‐based materials.

## 2 MATERIALS AND METHODS

### 2.1 Materials processing and characterization

The samples of pure Zn, Zn‐3Ag, and Zn‐3Ag‐0.5Mg (wt %) alloys were prepared using pure elements supplied by Onyxmet: zinc (99.995 wt %), silver (99.995 wt %), and magnesium (99.95 wt %). The ingots were fabricated by means of induction melting at 650°C and gravity casting into a steel mold. As‐cast Zn alloys were plastically deformed at 200°C and subsequently subjected to several passages of cold rolling at RT to obtain a uniform, equiaxed, fine‐grained microstructure. Microstructural observations were performed using a scanning electron microscope (SEM; FEI VERSA 3D) on samples' cross‐sections prepared by standard metallographic procedures described in details in our previous work.^15^ The chemical composition was analyzed using a wavelength dispersive X‐ray fluorescence spectrometer (WD‐XRF; Rigaku ZSX Primus IV). The signal was collected from an area of 3.14 cm^2^ and the sample was rotated at 30 rpm during the measurement. Additionally, the phase composition was determined via X‐ray diffraction measurements using an X‐ray diffractometer (XRD; Panalytical Empyrean) with Co‐K_α_ radiation (λ = 1.789 Å). Diffractograms were collected with a scanning rate of 0.4°/min and a step size of 0.02°. For XRD spectra analysis, the ICSD database was taken as a reference.

### 2.2 Cytotoxicity tests

#### 2.2.1 Cell culture of osteoblast‐like MG‐63 osteoblast‐like cell line and specimen's preparation

The in vitro cytocompatibility evaluation of Zn and Zn‐3Ag, Zn‐3Ag‐0.5Mg alloys was performed using direct and indirect methods on human osteosarcoma cells (MG‐63; Sigma‐Aldrich). Tests were performed following the ISO 10993 standard. All samples were prepared as flat disks with the diameter of 10 mm and thickness of 1 mm, polished with grinding papers up to #4000 grit, ultrasonically cleaned with ethanol, and then sterilized at 160°C for 2 h for the tests.

Cells were cultured in Dulbecco's modified Eagle's medium (DMEM; Gibco) supplemented with 10 vol % of fetal bovine serum (FBS; Corning) and 1 vol % of penicillin–streptomycin (PenStrep; ThermoFisher), which will be further referred to as the cell culture medium (CCM). Cells were incubated at 37°C in a humidified atmosphere (95%) containing 5% CO_2_. During the preparation of MG‐63 cell cultures, the distribution and morphology of cells were observed using light microscopy (Primovert, Carl Zeiss) to evaluate their development over time and determine their final cell density.

#### 2.2.2 Direct method of in vitro cytotoxicity assay

For the direct cell assay, 500 μl of MG‐63 cell droplets with 5 × 10^4^ cells per well were seeded on the flat surface of the sterilized disk samples and incubated at 37°C and 5% CO_2_ for 24 h. For each cytotoxicity assay, five samples of pure Zn, Zn‐3Ag and Zn‐3Ag‐0.5Mg alloys were tested. After incubation, the cell viability was measured based on the transition of tetrazolium salt into formazan by intracellular enzymes. For this purpose, the solution composed of 1% of WST‐8 (Sigma‐Aldrich) reagent and 99% of fresh complete medium was prepared. After removing the old CCM, washing the cells with phosphate buffered saline (PBS), 500 μl of WST‐8 solution was added to the wells, and the well‐plates were placed into the culture incubator for ~4 h at 37°C and 5% CO_2_. Next, 100 μl of the solution was transferred from each well to a 96‐well plate. The absorbance was measured at 450 nm using a UV–Vis spectrometer (FLUOstar Omega microplate reader, BMG Labtech). The relative cell viability was calculated as follows (OD—optical density):

(1) $$\text{Relative cell viability}\;\left(\%\right)=\frac{\text{sampl}e_{\mathrm{OD}}-{\text{blank}}_{\mathrm{OD}}}{\text{contro}l_{\mathrm{OD}}-{\text{blank}}_{\mathrm{OD}}}\;x\;100\%.$$

The same WST‐8 assay procedure was used for the solution taken from samples immersed in CCM for 24 h, however, without any seeded cells to check if there is any interference of released metallic ions and tetrazolium salts to formazan conversion without cells assistance. The results were then presented as a mean value and standard deviation of 5 replicas of each material.

The next step after the cell viability WST‐8 assay was analyzing the lactate dehydrogenase (LDH) assay to correlate the number of cells. The LDH release level was measured after 1‐day of incubation using an LDH‐activity quantification kit (TOX7, Sigma‐Aldrich). The cells were carefully washed with PBS and then lysed using a lysis buffer (0.1 wt % Triton X, 20 mM TRIS, 1 mM MgCl_2_ and 0.1 mM ZnCl_2_). Following the TOX‐7 protocol, a master mix (LDH‐MM) (20 μl of dye solution, 20 μl of cofactor, 20 μl of substrate solution) was prepared. Afterwards, 140 μl of the lysate was mixed with 60 μl of LDH master mix (LDH‐MM) and incubated at RT for 30 min, during which reduced nicotinamide‐adenine‐dinucleotide (NADH) transforms tetrazolium salt to formazan. After this time, the enzymatic reaction was stopped by adding 300 μl of HCl (1 M). A UV–Vis spectrometer (FLUOstar Omega microplate reader, BMG Labtech) was used to measure the absorbance at 490 and 690 nm (background absorbance) and must be subtracted from the primary OD values at 490 nm. The total amount of LDH release was quantified in relation to lysis control. The results were then presented as a mean value and standard deviation of 5 replicas of each material.

The cells' morphology and distribution on the samples' surfaces were analyzed using an SEM‐EDS system (Merlin Gemini II, ZEISS) equipped with energy dispersive X‐ray spectrometry (EDS, QUANTAX, Bruker). Prior to analysis, three samples of each material were prepared by removing the old CCM and washing them with PBS. Then, the PBS was replaced by two fixing solutions, the first containing glutaraldehyde and the second additionally containing paraformaldehyde, each for 1 h. Next, the samples were dehydrated to remove water using ethanol/distilled water gradient solutions (30%, 50%, 70%, 80%, 90%, 95%, and 100%) for 30 min each. After the final dehydration step, the samples were submerged in fresh ethanol and placed in a critical point dryer (EM CPD300, Leica). The final step, before SEM imaging, was coating the samples with 10 nm of gold by a turbo‐pumped sputter coater (Q150T, Quorum Technologies, Laughton).

#### 2.2.3 Indirect method of in vitro cytotoxicity assay

The extracts for the indirect cell assay were prepared by placing the disk samples in CCM for 24 h in a cell culture incubator at a controlled temperature of 37°C and 5% CO_2_ at the ratio of 1.25 cm^2^/ml, as recommended by the ISO 10993‐5: 2009 standard. Next, the supernatant was collected and diluted to obtain the following extract concentrations: 100%, 50%, 25%, 12.5%, 5% for further analyses.

In parallel, the MG‐63 cells were seeded into the 96‐well plate with 1 × 10^4^ cells per well and cultured for 24 h. After incubation, the original culture medium was removed from the wells, and the cells were placed into contact with the diluted extracts and then incubated for a further 24 h at 37°C and 5% CO_2_. 100 μl of the culture medium with 1 × 10^4^ cells was taken as a positive reference.

Afterwards, cell viability was assessed quantitatively using WST‐8 and LDH assays. Analogous procedures were used as during direct cell culture tests. Five replicas of all prepared extracts from pure Zn, Zn‐3Ag, Zn‐3Ag‐0.5Mg alloys were used per each colorimetric assay.

The cytotoxicity assays were combined with morphology and cell distribution microscopic observations. Live staining with green Calcein AM (calcein acetoxymethyl ester, Invitrogen) and blue DAPI (4′,6‐diamidino‐2‐phenylindole, dilactate, Invitrogen) was performed to image the cells' cytoplasm and nucleus, respectively. First, the CCM was removed and the samples were cleaned with PBS. A master mix, containing 4 μl Calcein‐solution in 1 ml PBS, was added and the samples were incubated for 45 min. Next, the solution was removed, the samples were washed with PBS, and the cells on the surface were fixed using a fixing solution (3.7% Paraformaldehyde in PBS) for 15 min. Afterwards, the solution was removed and the samples were rewashed using PBS. A second master mix, comprised of 1 μl DAPI‐solution in 1 ml PBS, was added and the samples were incubated at RT for 5 min. After removing the staining solution, the samples were kept in PBS before imaging using a fluorescence microscope (Axio Observer, Carl Zeiss, Jena).

### 2.3 Wettability and roughness

Advancing contact angles (CAs) were measured on polished samples by pipetting 3 μl volume droplets of deionized water in five spots on each sample. Immediately after droplet placement, images were taken using an EOS 700D camera (Canon, Japan). The advanced CA was measured using ImageJ (NIH, USA) software. The average CA was determined based on images of five droplets deposited on representative samples of Zn, Zn‐3Ag and Zn‐3Ag‐0.5Mg. Experiments were carried out at a temperature of 24°C and a humidity of 43%.

An atomic force microscope (AFM, CoreAFM, Nanosurf) was used to measure the surface roughness of polished samples. Roughness measurements (R_a_) were carried out in contact mode perpendicular to the scanning direction with a constant force of 0.18 Nm^−1^. All measurements were carried out with a scan rate of 1.5 Hz. Data processing was carried out using Gwyddion open‐source software. The average R_a_ values were calculated based on 10 different measurements performed on one sample representative for each material.

### 2.4 Ion concentration, corrosion rate and pH measurements

Extract (Section 2.2.3) ion concentration was determined via inductively coupled plasma–atomic emission spectroscopy (ICP‐AES; SPECTRO CIROS‐CCP). The extracts were diluted five times with distilled water before analysis. The Zn^2+^ concentration was determined using the emission line at 213.856 nm. The Ag^+^ and Mg^2+^ concentrations were below the detection limit of the ICP‐AES and could not be quantified. All experiments were conducted in triplicate.

The estimated corrosion rate (C_R_) was calculated based on the determined Zn^2+^ ion release in 100% extracts after 1‐day immersion of metallic disks in CCM, according to the following formula^30^:

(2) $$C_{R}=\frac{C\cdot V}{S\cdot T},$$

where $C_{R}$ is given in μg/cm^2^/day; C is the amount of released ions (μg/ml); V is the solution volume (ml); S is the sample surface area (cm^2^); T is the incubation time (days).

Additionally, the pH of the culture medium collected from the wells after 24 h of incubation was measured at 25°C using a pH meter (FiveEasy pH meter F20, Mettler Toledo). The pH results were averaged from three measurements per each material.

### 2.5 In vitro antibacterial properties assessment

#### 2.5.1 Preparation of bacteria

*S. aureus* (ATCC 25923) and *E. coli* (ATCC 25922) were used as microorganism strains for antibacterial assays. Lysogeny broth (LB) medium (Luria/Miller) was purchased from Carl Roth GmbH (Karlsruhe, Germany).

#### 2.5.2 Determination of antibacterial activity by turbidity assay

The antibacterial activity of the Zn‐based samples and Grade 2 titanium (control sample) was determined using *S. aureus* (Gram‐positive) and *E. coli* (Gram‐negative) bacteria strains. First, both bacteria strains were cultivated in LB medium at 37°C for 24 h. Subsequently, the inoculated bacterial suspension's OD value (Thermo Scientific GENESYS 30) was set to 0.015. Next, the sterilized samples were immersed in the prepared suspension and incubated for 3, 6, and 24 h at 37°C. At each time point, the OD of the samples (100 μl) was measured at 600 nm using a spectrophotometric plate reader (PHOmo, Anthos Mikrosysteme GmbH). Finally, the relative bacterial viability was calculated using the following Equation (3):

(3) $$\text{Relative bacterial viability}\;\left(\%\right)=\frac{\text{sampl}e_{\mathrm{OD}}-\text{blank}}{\text{contro}l_{\mathrm{OD}}-\text{blank}}\;x\;100\%.$$

LB media with and without bacterial strains were used as a control reference and blank, respectively. Each sample was carried out in triplicate.

#### 2.5.3 Determination of antibacterial activity by agar diffusion

The antibacterial activity was also assessed via a direct agar diffusion test. It was performed and analyzed according to the protocol described elsewhere.^27^ After 16 h of incubation, the inhibition of bacterial growth was determined based on measuring the inhibition zone according to the ISO 20645:2004 standard. The quantitative results were averaged out from three samples for each material.

### 2.6 Statistical analysis

All measured data were expressed as mean ± SD. If not stated otherwise, graphs display average values with uncertainty bars representing the standard deviation of the means calculated for at least three samples or replicates for each material. The statistical analysis was performed by means of analysis of variance (ANOVA) and Bonferroni's test using the OriginPro software. The statistically significant difference between groups was classified using a *p*‐value determined as *p* < .05.

## 3 RESULTS

### 3.1 Microstructure

**FIGURE 1:**
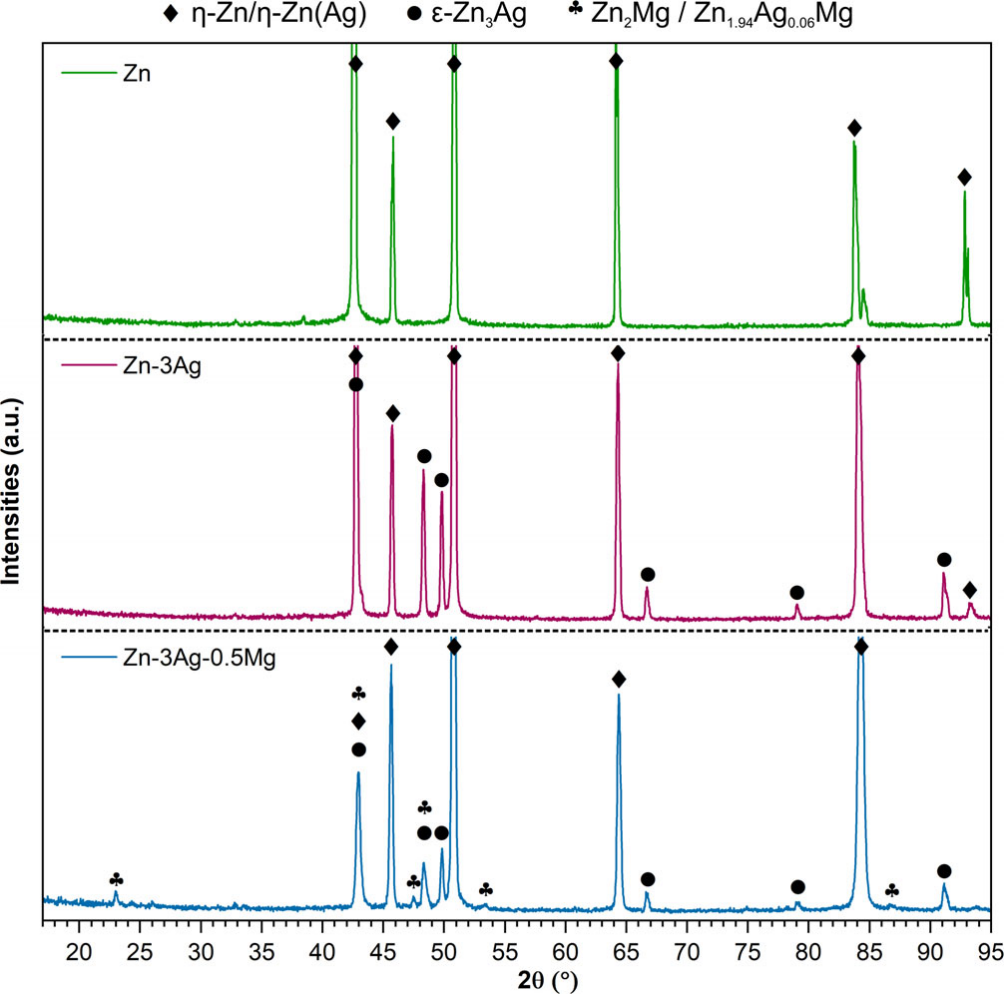
X‐ray diffraction patterns showing phase composition of pure Zn, Zn‐3Ag, and Zn‐3Ag‐0.5Mg alloys

According to the WD‐XRF analysis presented in Table 1, the concentration of alloying additions in the fabricated alloys is close to the nominal chemical composition. The qualitative phase composition analysis was performed based on the acquired X‐ray diffraction patterns shown in Figure 1. As it can be seen, the microstructure of the Zn‐3Ag alloy is composed of the η‐Zn phase, most likely enriched in Ag and intermetallic ε‐Zn_3_Ag phase. In the Zn‐3Ag‐0.5Mg alloy, besides those two phases, also the Zn_2_Mg phase, with possible Ag enrichment, was identified in the spectrum.

**TABLE 1:** Chemical composition (wt %) of Zn and Zn alloys measured by the WD‐XRF

	Zn (wt %)	Ag (wt %)	Mg (wt %)
Zn	100	–	–
Zn‐3Ag	96.71	3.29	–
Zn‐3Ag‐0.5Mg	96.17	3.36	0.47

**FIGURE 2:**
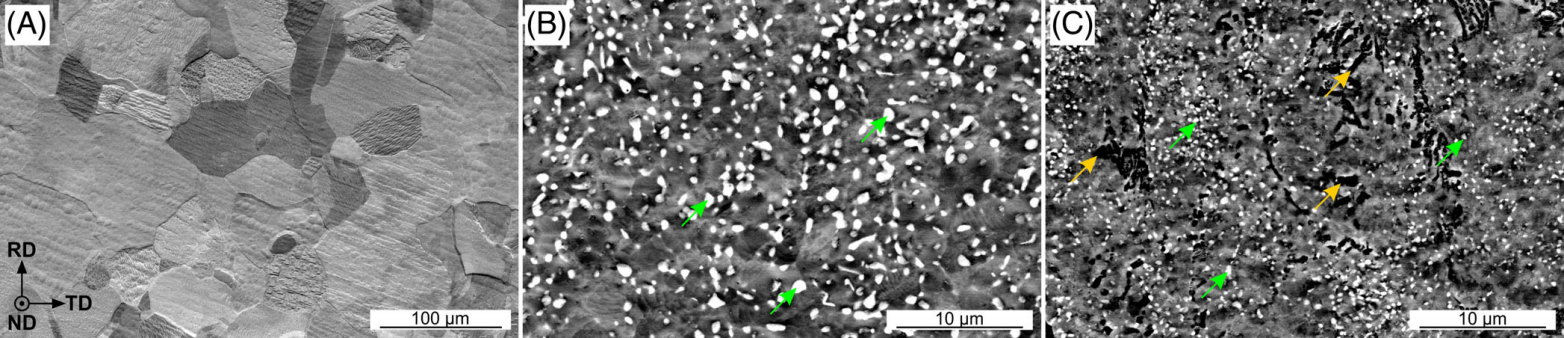
Microstructures of cold‐rolled pure Zn (A) and Zn‐3Ag (B), Zn‐3Ag‐0.5Mg (C) alloys in the longitudinal cross‐section (RD–rolling direction, TD–transverse direction, ND–normal direction). Note the different scale bars in the figures of pure Zn and Zn alloys. Green arrows indicate Ag‐rich precipitates and yellow arrows indicate Mg‐rich precipitates

Figure 2 shows SEM images of the microstructure of pure Zn (Figure 2A), as well as the Zn‐3Ag (Figure 2B) and Zn‐3Ag‐0.5Mg (Figure 2C) alloys. The microstructure of Zn is composed of recrystallized equiaxed grains, after the applied plastic deformation process resulted in relatively large grain size. White precipitates marked with green arrows in Figure 2B,C belong to the ε‐Zn_3_Ag phase. In the Zn‐3Ag‐0.5Mg alloy, the precipitates visible as black zones and marked with yellow arrows are enriched in Mg and, according to presented XRD, are Zn_2_Mg or Zn_1.94_Ag_0.06_Mg phases, which is consistent with our previous studies.^15^, ^27^, ^31^ It is clear that alloying additions refine the grain size and contribute to the formation of a multiphase microstructure. Such refined microstructures can benefit the uniform biodegradation process in physiological solutions, which is exceptionally important as excessive localized corrosion may lead to catastrophic loss in mechanical integrity of implants being in contact with damaged tissue.^10^

### 3.2 Direct contact cytotoxicity in vitro assay

**FIGURE 3:**
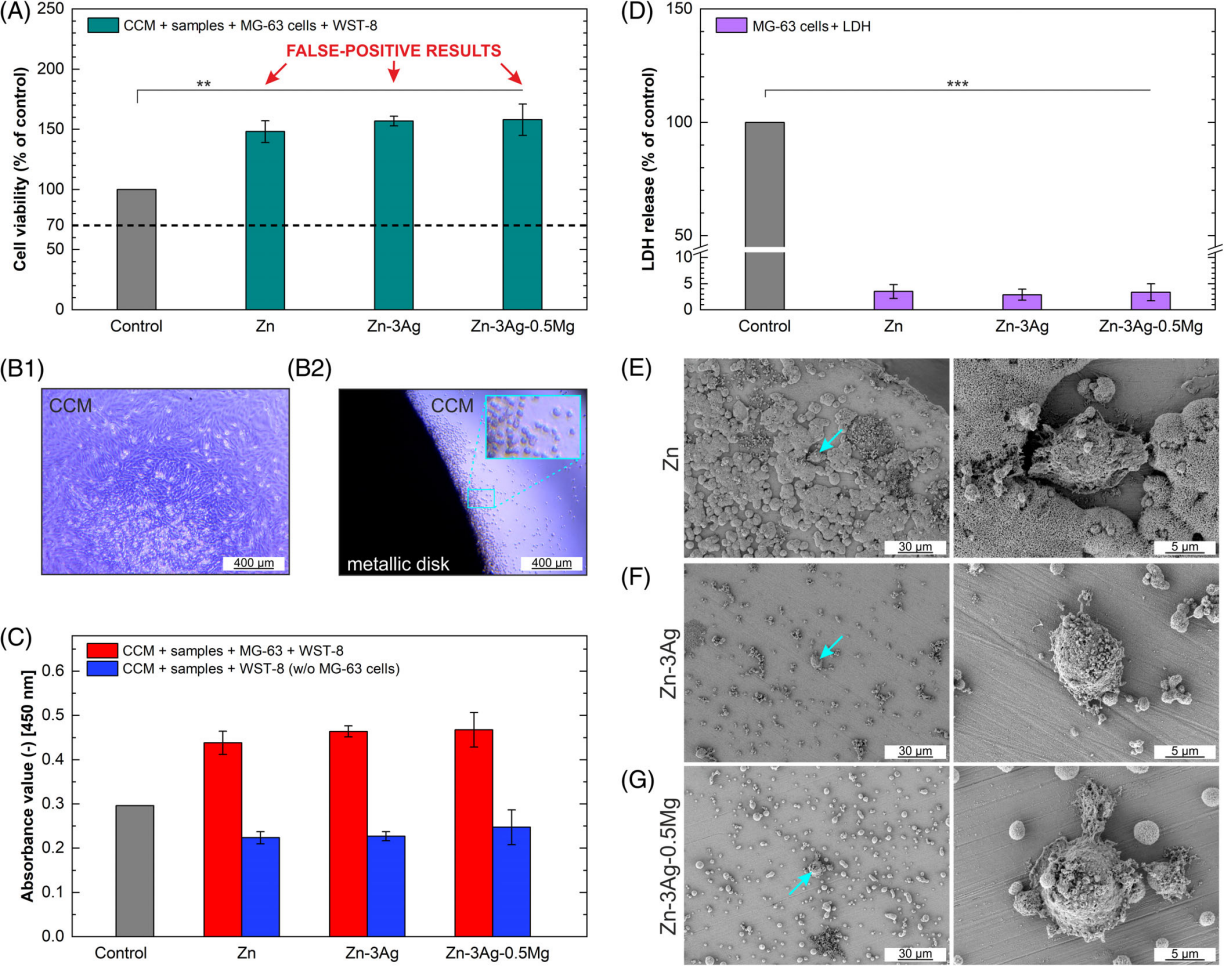
Cytocompatibility of pure Zn, Zn‐3Ag, and Zn‐3Ag‐0.5Mg alloys. Cell viability of MG‐63 cells after direct 1‐day incubation on Zn‐based metallic disks (A). Microscopic images of living cells in control sample without Zn‐based disk (B1) and dead cells around Zn‐based disk (pure Zn was shown as a representative example for all Zn‐based disks) (B2). Comparison of the absorbance value measured using spectrophotometer at 450 nm after 4‐h incubation of Zn‐based materials' discs with the WST‐8 reagent added to the CCM with and without MG‐63 cells (C). Control: Fresh complete medium + MG‐63 cells + WST‐8. LDH release level for MG‐63 cells after direct 1‐day incubation on Zn‐based metallic disks (D). SEM images of adhered single cells and corrosion products observed on the discs' surface of pure Zn (E), Zn‐3Ag (F), Zn‐3Ag‐0.5Mg (G) after 1‐day incubation with MG‐63 cells. All results given in graphs as mean ± SD (**p < .01, ***p < .001)

The in vitro cytocompatibility was evaluated based on the direct incubation of MG‐63 cells on flat surfaces of the examined Zn‐based materials. The WST‐8 and LDH colorimetric assays were performed to obtain quantitative information about cell viability. According to data presented in Figure 3A, the viability of MG‐63 cells after 1‐day incubation assessed in direct contact with Zn metallic disks was extremely high. Observations via light microscope revealed that in a control sample without a metallic disk inside the well (Figure 3B1), there was a high cell density, with the cells being large in size and possessing a spread morphology, while almost all cells that were in contact with pure Zn (Figure 3B2) and Zn alloy disks were round shape and small. Additionally, trypan blue staining (not presented here) confirmed the occurrence of dead cells that had been in contact with Zn‐based materials (dark blue coloring), and highly‐dense living bright cells on the control sample.

Due to these divergent results, the WST‐8 assay was repeated for the same samples without cells. In general, the NADH coenzyme and dehydrogenases from metabolically active cells reduce tetrazolium salts to intensely colored formazan products (a strong orange dye with an absorption maximum at 450 nm), which are quantified by absorbance measurements. The comparison between the measured absorbance values of both variants (with and without cells), included in Figure 3C, indicates that Zn^2+^ ions undoubtedly interfered with the WTS‐8 reagent. Furthermore, the OD measured in the range of 0.217 to 0.243 for Zn‐based disks without cells is close to the value obtained for the reference control sample with cells (0.296), which means that Zn‐based samples react with tetrazolium salts and contribute to formazan formation resulting in the CCM color change.

As the direct cytotoxicity tests using WST‐8 exhibited inconsistencies at high Zn^2+^ ion concentrations, measurements of LDH release were performed as an additional readout for cytotoxicity. In the LDH assay low absorbance values were measured, indicating cytotoxicity of Zn‐based samples. The relative LDH release level presented in Figure 3D does not exceed 5% for the investigated samples, indicating a small amount of viable MG‐63 cells after 1‐day direct tests. No significant difference between the cytotoxicity of the tested samples was recorded. It is consistent with microscopic observations of dead cells present on the disks' surface.

According to SEM images shown in Figure 3E–G, immersion in CCM and/or the use of a cell fixing procedure results in the formation of corrosion products, among which few cells were found. The morphology of small, spherical, almost unadhered to the surface cells indicates cell death. On pure Zn and Zn‐3Ag alloys, only single cells were present on the surface, while the number of cells on the surface of the Zn‐3Ag‐0.5Mg alloys was slightly higher.

**FIGURE 4:**
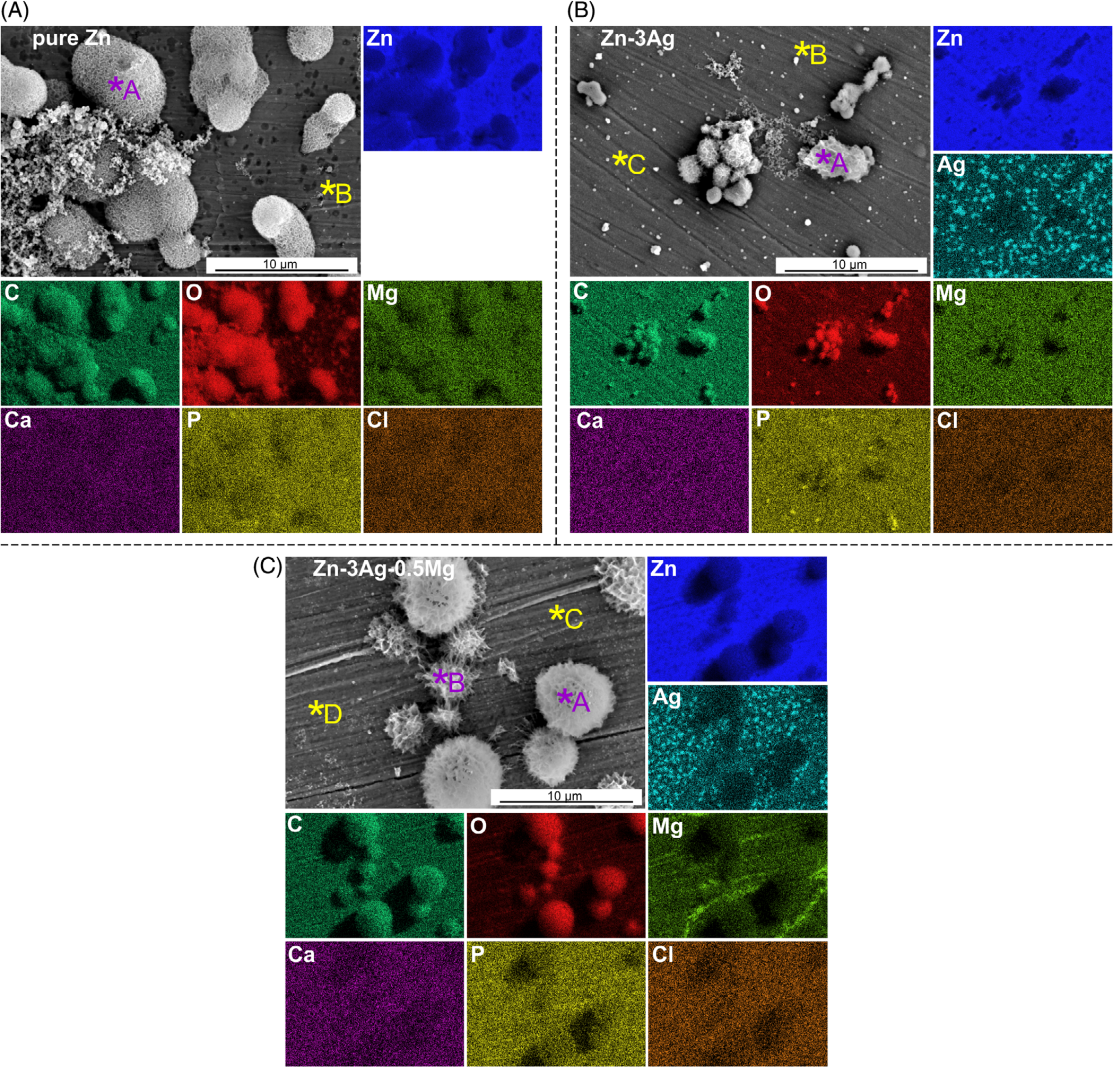
SEM images of surface morphology and EDS elemental maps (for Zn, Ag, Mg, C, O, Ca, P, Cl) for Zn‐based materials' disks and corrosion products formed on the surface after direct 1‐day incubation, with MG‐63 cells and SEM cells' fixation procedure; pure Zn (A); Zn‐3Ag (B); Zn‐3Ag‐0.5Mg (C). Points A–D marked with asterisks are the spots for EDS point microchemical analysis, which results are shown in Table 2. EDS, energy dispersive X‐ray spectrometry; SEM, scanning electron microscope

SEM‐EDS analysis revealed the chemical composition of the formed corrosion products on the surface after incubation of Zn‐based samples with cells. According to the elemental distribution maps presented in Figure 4, it can be seen that the corrosion products have a size of a few micrometers. They are globular, and some of them have a more developed needle‐like morphology. EDS point analysis results, summarized in Table 2, indicate that the corrosion products are composed mainly of Zn, O, C, with trace amounts of P. EDS spectra collected from the “bare” metal area revealed a thin surface layer consisting of O and C and small amounts of Cl and Ca (not including the elements comprising the sample). Evident differences in Ag and Mg concentration in the Zn‐3Ag and Zn‐3Ag‐0.5Mg alloys result from the presence of Ag‐rich particles present within the electron beam interaction volume during EDS analysis.

**TABLE 2:** EDS point analysis of corrosion products and samples' surface corresponding to the marked asterisks in Figure 4

Sample	EDS point analysis	Element (wt %)							
		Zn	Ag	C	O	Mg	P	Cl	Ca
Pure Zn	*A	67.90	–	12.60	19.12	0.11	–	0.12	0.17
	*B	90.71	–	7.16	2.08	0.03	–	0.03	0.02
Zn‐3Ag	*A	58.43	0.23	20.58	19.93	0.04	0.59	0.03	0.19
	*B	9.98	3.67	0.01	0.42	0.05	0.15	70.89	14.83
	*C	10.67	3.02	0.08	0.48	0.09	0.24	83.96	1.47
Zn‐3Ag‐0.5Mg	*A	62.53	0.04	14.66	22.33	0.13	0.07	0.08	0.19
	*B	61.96	0.57	15.92	20.39	0.24	0.69	0.05	0.20
	*C	81.85	5.80	8.56	2.97	0.40	0.25	0.05	0.13
	*D	76.12	9.42	8.94	4.12	0.73	0.44	–	0.23

### 3.3 Wettability, roughness, and pH measurements

**FIGURE 5:**
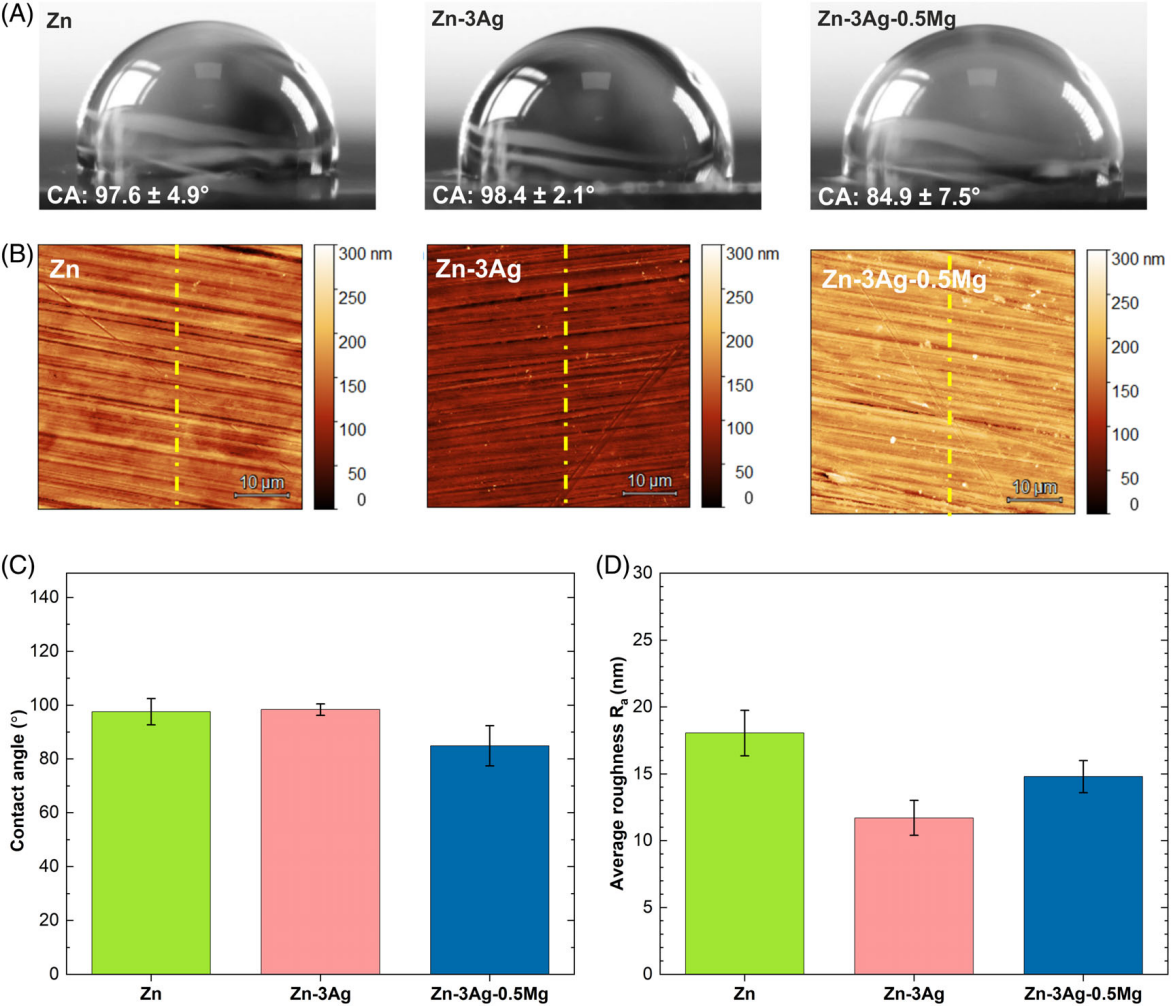
Optical images showing a droplet on Zn‐based metallic disks' surface for the contact angle measurements (A). AFM maps showing the surface roughness (B) of pure Zn, Zn‐3Ag, Zn‐3Ag‐0.5Mg alloys. Graphs showing the mean values with SD of the contact angle (C) and roughness Ra (D)

The pH after the experiment was measured to find a reason for the cytotoxicity observed for Zn‐based disks against MG‐63 cells. Additionally, the wettability and roughness of the prepared samples were determined. In principle, a smaller CA, high surface roughness, and high surface energy lead to better cell adhesion on the material's surface.^32^ As is well known, a material is classified as hydrophilic when the CA is <90°, and as hydrophobic when >90°. The measurements presented in Figure 5A,C revealed that the Zn‐3Ag‐0.5Mg alloy exhibits the lowest CA value, which amounted to 84.9 ± 7.5°. No significant difference between CAs was noted for pure Zn and the Zn‐3Ag alloy. However, they had lower wettability and were more hydrophobic than the Zn‐3Ag‐0.5Mg alloy since the CA reached 97.6 ± 4.9° and 98.4 ± 2.1°, respectively.

The average roughness (R_a_) values for Zn‐based materials (Figure 5B,D) are in ascending order: Zn‐3Ag: 11.7 ± 1.3 nm, Zn‐3Ag‐0.5Mg: 14.8 ± 1.2 nm, and Zn: 18.1 ± 1.7 nm. Despite evident differences in R_a_ values, the samples' roughness is the same order of magnitude, which in terms of cytotoxicity evaluation makes these differences insignificant.

The pH of CCM used for the experiments was 7.82 ± 0.01. After the direct cytotoxicity test, the pH measured for pure Zn, Zn‐3Ag, and Zn‐3Ag‐0.5Mg alloys was slightly higher at 7.88 ± 0.04, 7.85 ± 0.03, and 7.92 ± 0.03, respectively. As can be seen, the differences between the pH of the samples are not significant, and after 24‐h incubation, the pH remained below 8, so the pH was eliminated as a factor that influences cell death, at least the global value of the CCM. Nevertheless, the possibility of a local pH increases on the disks' surface, possibly affecting cell viability, could not be ruled out.

### 3.4 Indirect in vitro cytotoxicity test

**FIGURE 6:**
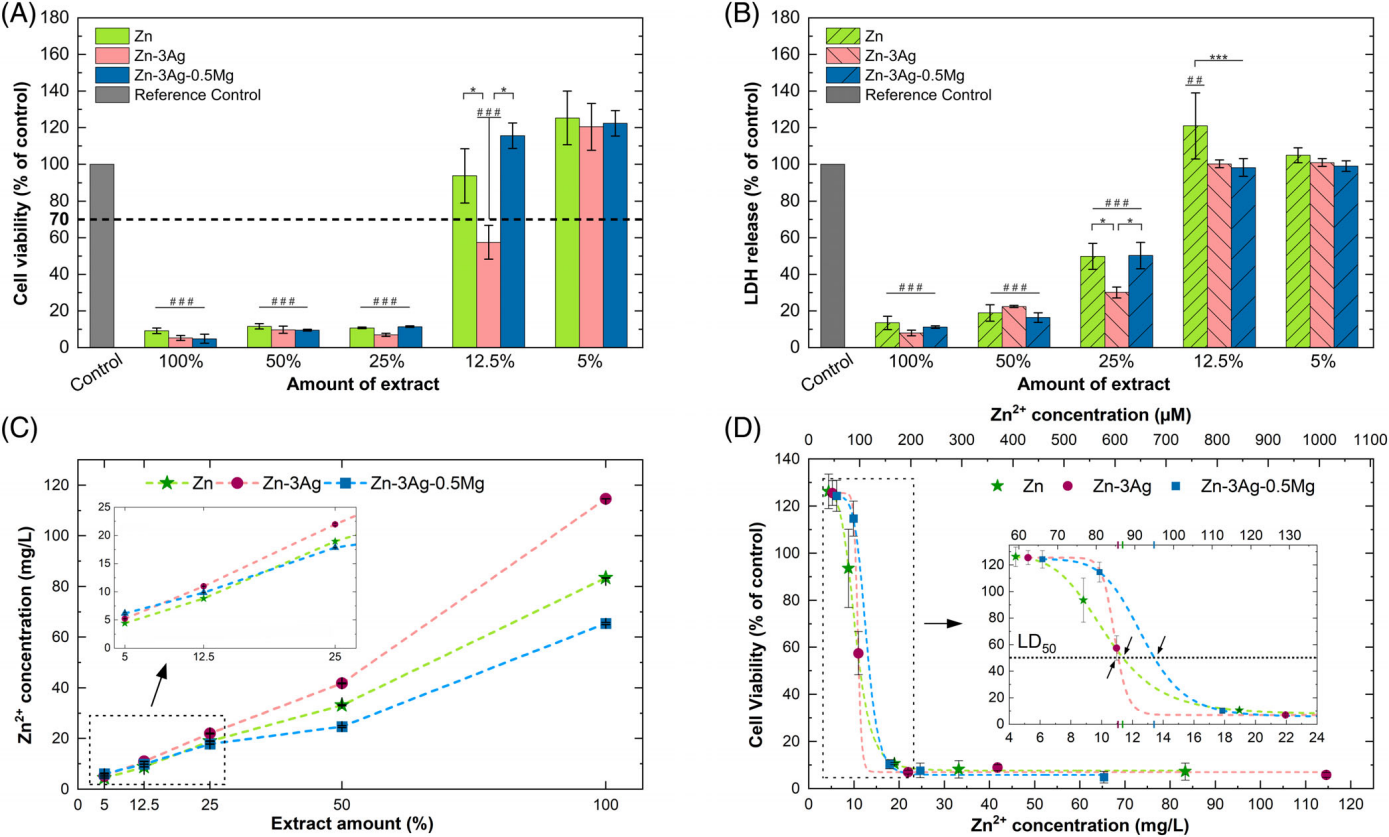
Viability of MG‐63 cells cultured indirectly in diluted extracts of pure Zn, Zn‐3Ag, and Zn‐3Ag‐0.5Mg (A). Lactate dehydrogenase release level after 1‐day co‐culture of extracts with MG‐63 cells (B). Zn^2+^ ions concentration in diluted extracts after 1‐day incubation (C). Variation of cell viability (for the WST‐8 assay) versus Zn^2+^ ions concentration in extracts (D). All results given as mean ± SD (*p < .05; **p < .01; ***p < .001 and ^##^ *p* < .01; ^###^ *p* < .001 compared to the reference control)

In Figure 6, cytotoxicity tests on the extracts prepared from pure Zn, Zn‐3Ag, Zn‐3Ag‐0.5Mg alloys were collected. The CCM without any extraction medium was used as a reference control. Cell viability was assessed using WST‐8 (Figure 6A) and LDH (Figure 6B) assays.

The cell viability increased with a growing dilution of the extract, which means that the MG‐63 cells were under proper conditions for proliferation. However, no significant differences between the samples were observed for 100% extracts, where all of them exhibited the cytotoxic effect. A sharp transition from toxic to nontoxic concentrations was noted between 25% and 12.5% extract of pure Zn and the Zn‐3Ag‐0.5Mg alloy, which seems to deliver a safe concentration of metallic ions for cell conditions. However, the Zn‐3Ag alloy shows a satisfactory level of cell viability (70%) only at 5% extract. No significant variations with alloying element additions in Zn alloys were noticeable in the WST‐8 assay for the tested extracts compared to pure Zn.

To validate the WST‐8 assay results and to compare to direct cytotoxicity tests, the LDH assay was used for indirect tests. The main benefit of supplementing the cytotoxicity tests by LDH colorimetric assay is that it can mark only living healthy cells with intact cell membranes. The presence of LDH‐MM in CCM is used for determining cellular cytotoxicity. By using the LDH assay kit, the enzymatic reaction of tetrazolium salt conversion to red‐colored formazan occurs. The results presented in Figure 6B stand in agreement with the WST‐8 assay results (Figure 6A). At a maximum of 12.5% extract, all samples demonstrated LDH release on the same level as the reference (control). A lower LDH level was observed for the Zn‐3Ag alloy at 25% extract, but besides these results, no other significant differences between the Zn‐based materials were noted.

The colorimetric assays were supported by the ion release evaluation in diluted extracts (Figure 6C). The ICP‐AES method allowed only to determine the Zn^2+^ ions as the amount of Ag and Mg in the alloys and, in turn, in the extracts was below the detection limit. Furthermore, the variation in cell viability (from WST‐8 assay) vs. ion concentration was plotted in Figure 6D to estimate the value of the median lethal dose (LD_50_). In the current research, the Zn^2+^ ion concentration in the prepared extracts ranges between ~4.4 mg/L for the 5% extract of pure Zn up to ~114.6 mg/L for the 100% extract of the Zn‐3Ag alloy. At the two highest extract dilutions, the amount of released ions is close to one another, however, above 12.5%, the highest amount of released Zn^2+^ ions was detected in further extracts of the Zn‐3Ag sample. The approximate LD_50_ value was 11.4 mg/L, 11.1 mg/L, and 13.4 mg/L for pure Zn, Zn‐3Ag, and Zn‐3Ag‐0.5Mg alloys, respectively, so the differences between several materials are not significant. At the lowest extract concentration (5%), the concentration of Zn^2+^ ions released from Zn‐based samples ranged from ~4.4 to 6.2 mg/L. Therefore, it can be stated that the alloying additions do not significantly change the kinetics of ion release from the examined samples after 24‐incubation, however, a slightly higher amount of released Zn^2+^ ions from Zn‐3Ag samples may explain a slightly higher cytotoxic effect. This result suggests that the cytotoxicity may not be related to the Ag ions themselves but may concern the degradation kinetics in CCM and Zn^2+^ ions release.

**FIGURE 7:**
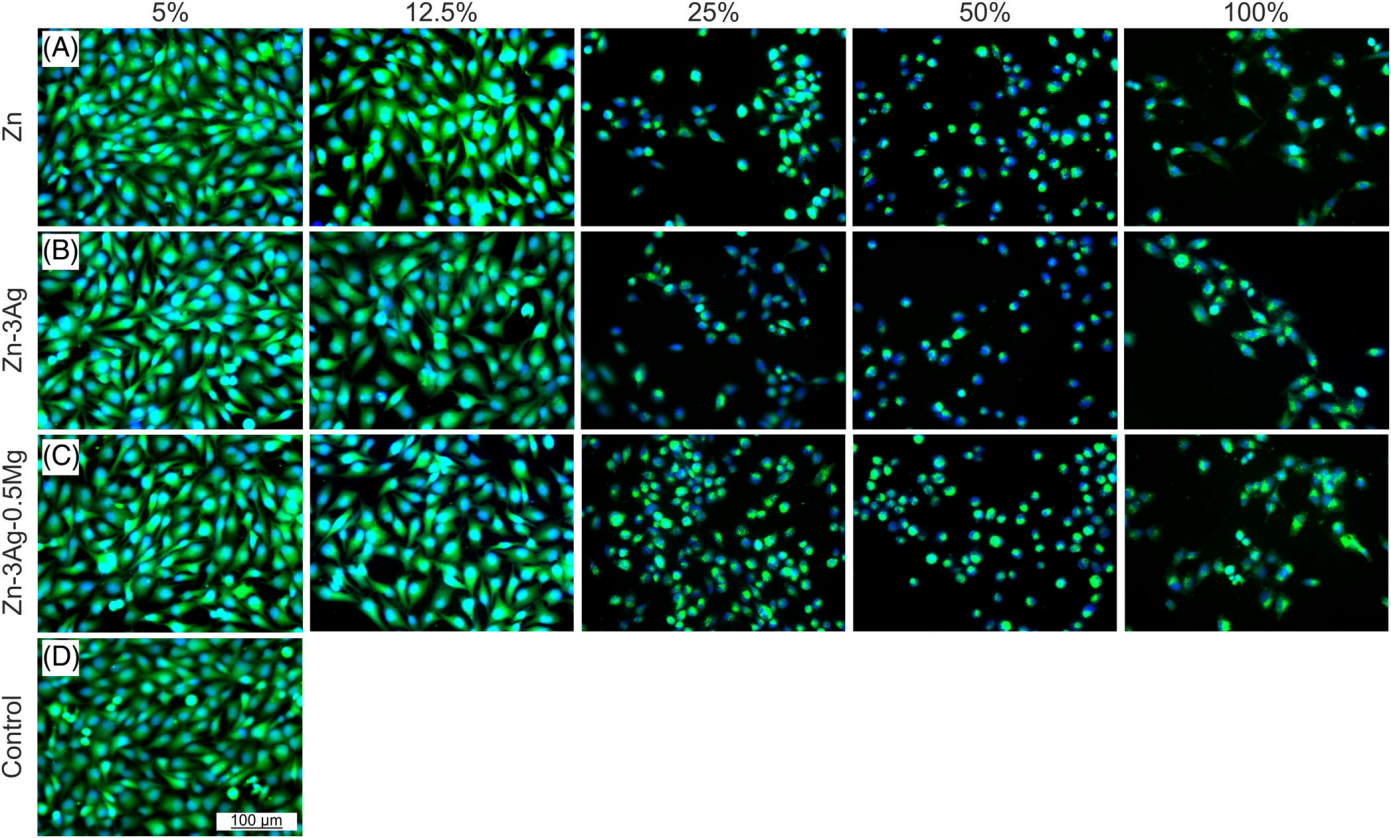
Fluorescent images showing the results of calcein‐DAPI staining of MG‐63 cells after 24 h of indirect culture for pure Zn (A), Zn‐3Ag (B), and Zn‐3Ag‐0.5Mg (C) alloys, and reference control (D). Cell nucleus: DAPI, cytoplasm: Calcein AM. Please, note that the scale bar, 100 μm, is the same for all presented images

Bearing in mind that the Zn^2+^ ions may interfere with tetrazolium salts, the fluorescent imaging of living cells in extracts was additionally performed and the results are included in Figure 7. The results are consistent with the quantitative results and confirm their reliability. For Zn‐based samples, it is clear that not only the number of cells grew with increasing extract dilution, so decreasing ion concentration, but also the cell morphology changed. Below 25% extract, cells were well developed. They are spread, have well‐defined cytoplasm (green color), and cover almost the entire available space in a well. High metallic ion concentration caused undesirable conditions for further cell growth and division. In this case, cells appear small and circular, with the cytoplasm at almost the same size as the cell nuclei.

**FIGURE 8:**
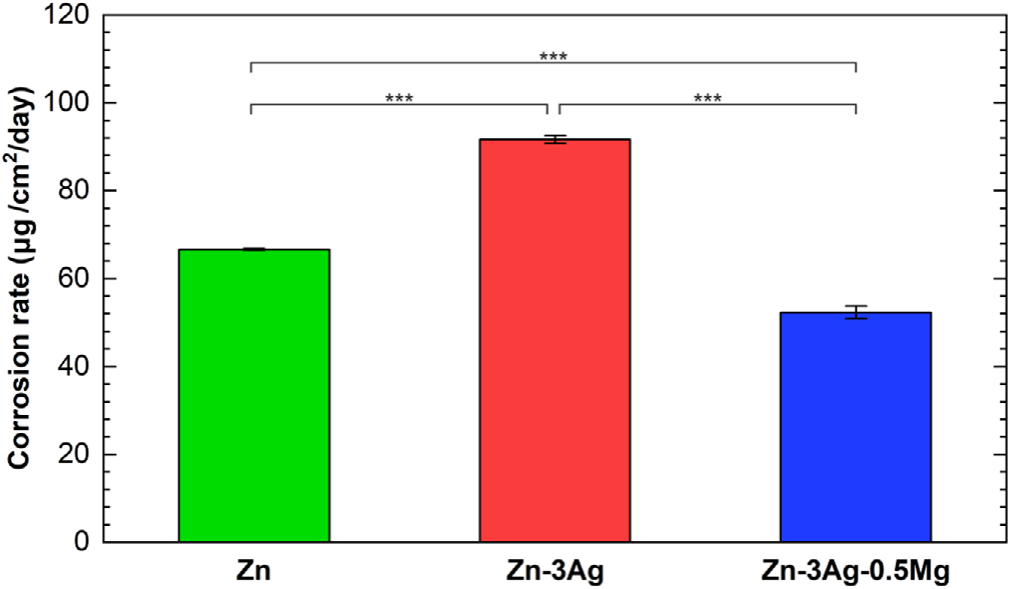
Corrosion rates of pure Zn, Zn‐3Ag, and Zn‐3Ag‐0.5Mg alloys immersed in CCM for 1 day determined based on the Zn^2+^ ions concentrations measured in the undiluted extracts. Results given as mean ± SD (***p < .001). CCM, cell culture medium

Ion concentration measurements allowed to estimate the corrosion rate corresponding to the amount of released Zn^2+^ ions in the undiluted extracts obtained after 1‐day immersion of the samples in the CCM. The results are presented in Figure 8. The corrosion rate of pure Zn amounted to 66.6 μg/cm^2^/day. It can be seen that the Zn‐3Ag alloy is the fastest corroding material at a rate of 91.6 μg/cm^2^/day, which is related to the highest concentration of released Zn^2+^ ions from the sample. According to other reports, ε‐Zn_3_Ag precipitates lead to the formation of micro‐galvanic cells with Zn matrix grains, which corrode at first because of the more cathodic electronegative potential.^17^, ^33^ In contrast, Mg additions resulted in slower corrosion at a rate of 52.3 μg/cm^2^/day, which is related to lower Zn^2+^ ions concentration. It has been reported that Mg‐rich precipitates corrode prior to the Zn matrix in the micro‐galvanic cells. Moreover, it might be related to the release of Mg^2+^ ions, which are incorporated in the corrosion layer and thus limit the material degradation process.^17^, ^33^

### 3.5 Antibacterial properties

**FIGURE 9:**
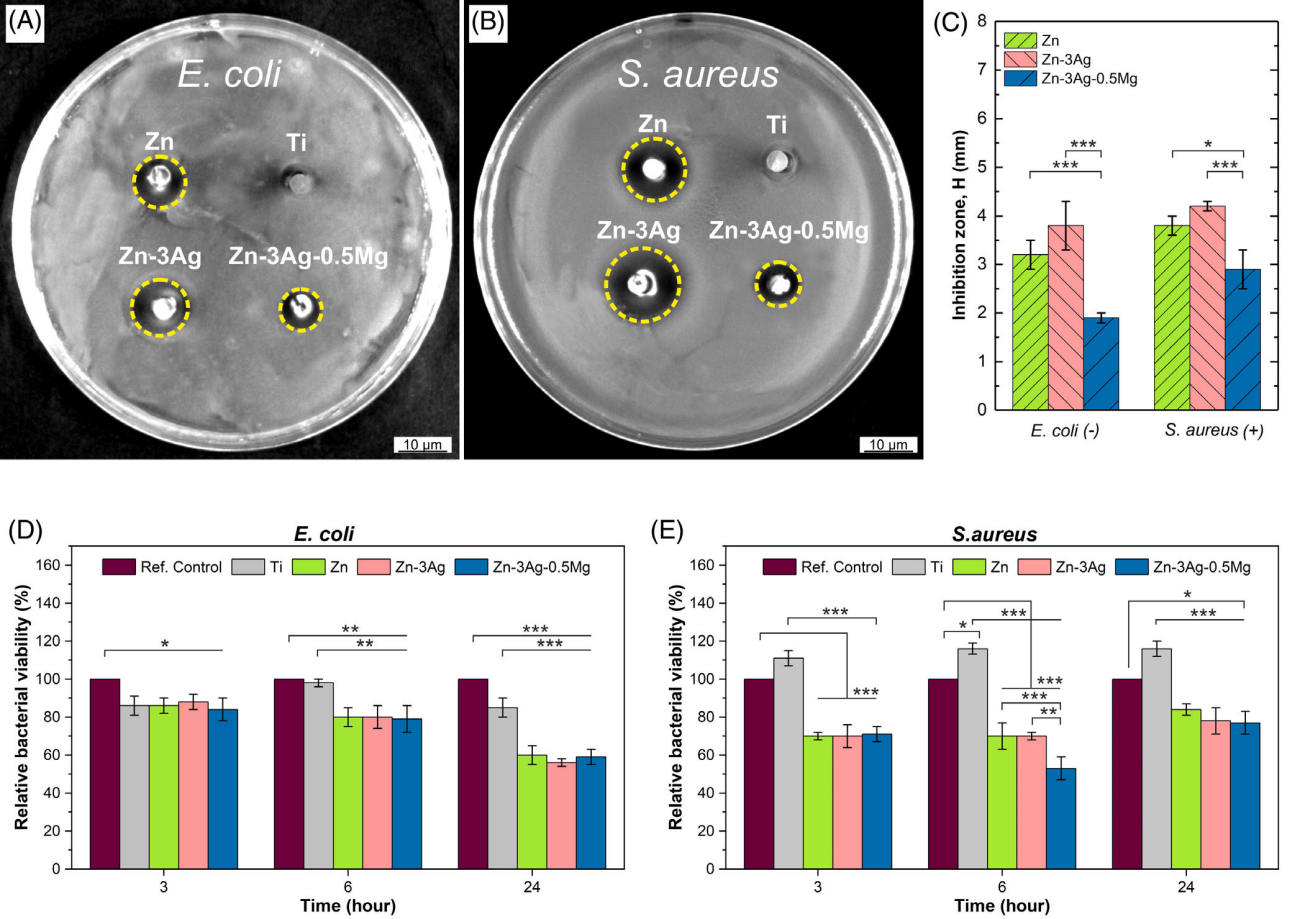
The digital photos of inhibition zone around Zn, Zn‐3Ag, Zn‐3Ag‐0.5Mg, and Ti samples for co‐cultured *E. coli* (A) and *S. aureus* (B) on Agar plates. The inhibition zone presented as an *H* parameter averaged from 3 plates' measurements (C). Relative *E. coli* (D) and *S. aureus* (E) bacteria viability in suspensions with the released metallic ions. All results given as mean ± SD (*p < .05; **p < .01; ***p < .001)

Figure 9 shows the antibacterial properties of Zn‐based samples. Agar well diffusion tests revealed the formation of an inhibition zone around pure Zn and Zn alloys against *E. coli* (gram‐negative) (Figure 9A) and *S. aureus* (gram‐positive) (Figure 9B). The inhibition of bacterial growth was observed for all Zn‐based samples. However, the effect was more substantial for pure Zn and the Zn‐3Ag alloy than for the Zn‐3Ag‐0.5Mg alloy against both types of bacteria. Visually, Ag additions caused slightly higher bacteria inhibition zone formation in the Zn‐3Ag alloy. However, the difference between the calculated average *H* value indicating the size of the inhibition zone (H > 1 mm means perfect antibacterial behavior) was statistically insignificant. Meanwhile, this *H* parameter (Figure 9C) was smaller by 41% against *E. coli* and 24% against *S. aureus* in the Zn‐3Ag‐0.5Mg alloy compared to pure Zn. Moreover, antibacterial activity depended on the analyzed bacterial strains, where the antibacterial effect against *S. aureus* was more noticeable than for *E. coli* by ~30%. No antibacterial effects were observed around Ti samples applied as a reference.

The antibacterial activity determined by direct turbidity indicated that the studied Zn‐based materials reduce the bacteria viability compared to Ti and reference samples, including only bacteria. Any discrepancies between the direct and indirect tests presented here may result from different sample diameters used for the experiment and thus slightly different ion release kinetics. Nevertheless, the OD results indicated that Zn, Zn‐3Ag, and Zn‐3Ag‐0.5Mg alloys have some antibacterial effect for both bacterial strains, which agrees with the Agar diffusion assay. The Zn‐based samples were more effective against *S. aureus* bacteria than *E. coli* (Figure 9D,E). However, there was no significant difference in their antibacterial effect between the pure Zn, Zn‐3Ag, and Zn‐3Ag‐0.5Mg alloys, except at 6 h incubation timepoint against *S. aureus* bacteria. After 6 h, the inhibition was higher for the Zn‐3Ag‐0.5Mg alloy than for the pure Zn and Zn‐3Ag alloy. Bacterial viability of 50% was observed for the Zn‐3Ag‐0.5Mg alloy for the 6 h timepoint against *S. aureus*, whereas the reduction of bacterial viability of *E. coli* was the strongest after 24 h for all Zn‐based materials. No significant difference between bacteria viability for pure Zn and the Zn‐3Ag sample was indicated in direct turbidity tests.

## 4 DISCUSSION

### 4.1 Considerations on the cytotoxic effect in Zn‐based materials

As a biodegradable implant material is introduced inside the human body and comes in contact with damaged tissue, the biological properties directly affect human life and health. Therefore, it is a matter of great importance that such materials should demonstrate indisputable biocompatibility and biosafety to avoid causing any harmful reactions to the host tissue by the implanted material and released corrosion products. As it has already been mentioned, in order to use Zn for load‐bearing biomedical applications, it is crucial to introduce alloying elements, which increase its mechanical properties and simultaneously do not affect its biodegradation behavior and biological properties.

Although, according to our studies, Zn‐based samples had a cytotoxic effect on MG‐63 cells in direct contact, no significant differences in the biological response to Zn alloys compared to pure Zn were found. Thus, it can be said that the cytotoxicity observed in the in vitro experiments for Zn alloys is not affected by the Ag and Mg additions, essential for demonstrating high strength and proper ductility. Some in vitro results have reported that Ag^+^ ions can contribute to the cytotoxic effect.^18^, ^34^, ^35^ However, a small amount of Ag, corresponding to the chemical composition of the investigated Zn alloys, is considered to be safe for living organisms.^23^, ^34^, ^36^ Since Mg^2+^ ions are known to govern a stimulating effect on new bone formation by increasing the proliferation and differentiation of osteogenic cells^25^ a positive effect of Mg^2+^ ions released from the sample on cytocompatibility was expected. However, in this case, the amount of Mg^2+^ ions might have been too small to reverse the high Zn^2+^ ions concentration‐induced toxicity. According to biological studies on the effect of co‐releasing Zn^2+^ and Mg^2+^ ions from Zn‐Mg composites, the amount of Zn^2+^ ions played a crucial role in the cytotoxic effect. The addition of Mg^2+^ ions (resulting in up to 27.9 μg/mL) had little effect on the cell morphology and their proliferation, while the twofold decrease in Zn^2+^ ions (from 23.9 to 12.1 μg/ml) provided high MC3T3‐E1 cell viability.^26^

In our studies, the samples' relatively high CA (~90°) and low surface roughness might also influence the lack of appropriate cell adhesion and cell growth in direct contact with the metallic disks. It was reported that the Mg‐based bulk metallic glass demonstrated higher viability (91.4 ± 0.3%) of MG‐63 cells cultured on samples with a higher surface roughness of 220 nm than on samples with a roughness of 70 nm (68.8 ± 0.2%).^37^ According to Reference 38, surfaces with R_a_ parameter of about 1–2 μm is optimal for osteoblastic cells. It means that the obtained surface roughness in this study (<20 nm) is far from the optimal value and might be the cause of poor cell attachment. The cytotoxic effect on MG‐63 cells was clearly visible when looking at the microscopic image (Figure 3B2) presenting dead cells in the well around the disk. SEM images showed that almost no cells could be found on the Zn and Zn‐3Ag disks' surface (Figure 3E‐F). A few more cells attached to the surface of the Zn‐3Ag‐0.5Mg alloy, which might be related to a smaller amount of released Zn^2+^ ions or to factors associated with the microstructure and surface chemistry of this material. Heterogeneities presented in fine‐grained multiphase Zn‐3Ag‐0.5Mg alloy are associated with multiple states having a local minimum of Gibbs energy, contributing to lower CA and more hydrophilic behavior of the surface liked by cells.^39^ Nevertheless, most of the cells found on the surface of the Zn‐3Ag‐0.5Mg alloy had an unhealthy, globular morphology suggesting that they were dead. These results may suggest that the cells died mainly due to the high local concentration of Zn^2+^ ions released from the samples. If this were the case, most of the cells had no chance to adhere to the samples' surface. The cytotoxicity may result from increased CCM osmolality due to high Zn^2+^ ions concentrations, imposing potential osmotic shock to the MG‐63 cells, leading to disrupted cell functioning and low survival rates.^40^, ^41^ A shift in pH toward an alkaline solution is not considered here as a factor affecting cell viability since the changes in pH were insignificant.

Results from SEM‐EDS observations (Figure 4 and Table 2) indicate that the released Zn^2+^ ions can react with ions included in the CMM, such as oxygen or carbonate ions, to form ZnO and ZnCO_3_ compounds. It is consistent with the elemental distribution maps and the enhanced Zn, O, and C concentrations detected in the corrosion products.^39^, ^42^ The same compounds were found during in vivo studies on pure Zn implanted in the form of wires into the arteries of rats and were not considered toxic.^11^ However, since the cytotoxicity of Zn‐based samples marked in static direct in vitro tests is related to the large amount of Zn^2+^ ions released upon the first contact with the CCM, it would be advantageous to control the degradation behavior and thus the ion release kinetics to create a much better environment for cells. According to literature reports, any pre‐treatment processes altering the Zn^2+^ ion release kinetics can positively influence cell viability.^8^, ^43^, ^44^, ^45^ It should be mentioned that based on the indirect studies, the cytotoxic effect was proved to be Zn^2+^ ion dose‐dependent, and extracts with lower Zn^2+^ ion concentrations demonstrated good cytocompatibility.

This brings to the fact that the clinical success of the biomedical application of Zn‐based materials depends on the amount of released Zn^2+^ ions necessary to stimulate cell growth and proliferation. From this perspective, Zn is alternatively considered as a beneficial element for bioactive glasses, that is, promising materials for repair and reconstruction of hard tissues or for surface modifications of Ti‐based orthopedic or dental implants. The incorporation of Zn^2+^ ions aims at facilitating better cells adhesion, bone osseointegration, mineralization, and antibacterial properties of Ti implants or bioactive glasses.^46^, ^47^, ^48^, ^49^ Although high Zn concentrations (10–20 μg/L) can be toxic to human dental pulp stem cells (hDPSCs) cells, low doses of Zn (0–5 μg/L) have good biosafety and can promote the proliferation and differentiation of hDPSCs cells.^50^, ^51^ Excellent in vitro and in vivo biological response including osteogenic activity and antibacterial activity was observed after Zn and Ag co‐implantation into Ti surface layers.^52^

### 4.2 The effect of Zn^2+^ ions and Ag, Mg additions on MG‐63 cell viability in extracts

Similar to the direct contact test, the incubation of MG‐63 cells with undiluted Zn‐based extracts induced a cytotoxic effect. Insufficient biocompatibility under in vitro conditions at high Zn^2+^ ion concentrations has been repeatedly reported in literature, but at the same time, cytotoxic reactions to Zn‐based materials has not been shown to be an issue during in vivo studies.^53^ The two main reasons that may explain the discrepancy between in vitro and in vivo conditions are the following: (1) the local concentration of excessively released metallic ions or degradation products is mitigated under dynamic conditions of the blood flow; (2) complex in vivo environment results in protein layer formation on the implant's surface, therefore inhibiting direct contact with the cells. Attempts to modify biodegradation and cytotoxicity tests have been undertaken to mimic in vivo conditions as closely as possible.^54^, ^55^, ^56^ The effect of the proteins included in the CCM should be considered, when comparing in vitro results to other studies performed in different simulated body fluids or under in vivo conditions. However, during direct comparison between the Zn‐based materials studied here, the proteins should not differently affect the biodegradation behavior of particular samples.^57^ In general, it has been reported that in the initial stage of degradation, albumins may adhere to the surface, cause the appearance of a passivation layer and slightly hinder the corrosion process.^56^, ^58^ Further immersion causes increased corrosion due to the dissolution of the metal matrix. However, if the immersion lasts long enough, the corrosion resistance may improve (up to 7 days), due to complex accumulation of the corrosion products and proteins on the sample's surface.^56^ Additionally, more uniform corrosion was observed in the protein‐containing physiological solutions.^58^ In the current studies, after 1‐day direct incubation of Zn‐based samples with cells, a corrosion product layer appeared on the surface, however, with an additional layer of adhered proteins not clearly visible. Therefore, 1‐day immersion in CCM might not be sufficiently long enough to form a compact protein‐based protection layer.

Typically used in vitro cytotoxicity test protocols for biodegradable Zn alloys may provide misleading results and overestimate the cytotoxic effect, when performed under static conditions, as they are prepared for non‐corroding metals. Thus, evaluating the Zn^2+^ ions' dose‐dependent cytotoxicity is more reliable in the case of the cellular response to biodegradable metals. By correlating quantitative cell viability results and ion concentration measurements in a series of extract dilutions, it is possible to estimate the acceptable limit value of Zn^2+^ ions released from the examined materials, below which the cytotoxic effect will be suppressed. A minimum 6‐fold dilution of extracts for indirect cytotoxicity testing specified in the ISO‐10993 standard has been recommended for Mg alloys^59^ and successfully transferred to Zn alloys.^60^ According to the ISO 10993 standard, if the cell viability (measured as a ratio to the positive control group) reduction is greater than 30%, the materials are considered cytotoxic. It means that materials with more than 70% cell viability demonstrate desired biocompatibility.

It can be concluded that the biological properties of the biodegradable materials directly correspond to their corrosion behavior. The concentration of ions released during the corrosion process can serve as an alternative parameter to the weight loss for corrosion rate calculations. After 1‐day of immersion in CCM, the corrosion rate ranged from 52.3 μg/cm^2^/day for the Zn‐3Ag‐0.5Mg alloy, up to 91.6 μg/cm^2^/day for the Zn‐3Ag alloy, with pure Zn's corrosion rate being in between. For comparison, research performed in other studies on pure Zn and Zn‐4Ag alloys in DMEM for 24 h revealed corrosion rates of 6.9 and 10.8 μg/cm^2^/day.^61^ 1‐day incubation of Zn‐Mg alloys in DMEM with 5% FBS showed that increasing Mg additions result in higher corrosion rates, with 13.4 μg/cm^2^/day reported for the Zn0.8Mg alloy^62^ and 52.0 μg/cm^2^/day for the Zn‐1.5Mg alloy.^8^ Therefore, the results of corrosion rate obtained in our studies are slightly higher than those presented for the Zn‐Ag and Zn‐Mg alloys. However, pure Zn in scaffold form was reported to corrode much faster at the rate of 433 μg/cm^2^/day, due to its porous structure and large surface area.^63^ The differences might result from the larger CCM volume used in our experiments for preparing the extracts for cytotoxicity tests and thus a higher amount of ions included in the CCM per 1 cm^2^ of the tested sample. The interaction of CCM with ions released from the Zn‐based material spontaneously forms corrosion products and accelerates the degradation process. According to the considerations in Reference 62, it is problematic to unambiguously define the acceptable corrosion rate, as it depends on the implant type, function, size, location in the body, and so forth. However, following these considerations and assuming a 5 mm diameter and 50 mm long fixation screw, with a surface area of 8 cm^2^, the Zn‐3Ag alloy with the highest corrosion rate (91.6 μm/cm^2^/day) would release approximately 0.7 mg of Zn^2+^ per day, with this value being even smaller for a screw made of the Zn‐3Ag‐0.5Mg alloy. This represents only a small contribution to the daily oral intake of Zn^2+^ that should not exceed 15–40 mg/day.^4^

It has already been presented that the safe ion concentration value for satisfactory cell viability is dependent on a few factors: (1) selected cell line; (2) used CCM that affects the corrosion process kinetics; (3) chemical composition and the state of the material.^54^, ^64^ For instance the LD_50_ value of Zn^2+^ ions can vary as follows: 50 μM (3.5 ppm), 70 μM (4.5 ppm), and 265 μM (17.5 ppm) (where ppm ≈ mg/L) for human dermal fibroblasts, human aortic smooth muscle cells, and human endothelial cells, respectively.^65^ Other studies demonstrated relatively high Zn^2+^ tolerance for the primary endothelial and smooth muscle cells that exhibit 50% survival at Zn^2+^ concentrations of 340 and 330 μM, respectively.^66^ It has also been reported that at low concentrations, Zn had no adverse effects on endothelial cell viability, while the amount of Zn^2+^ over 100 μM significantly decreased cell viability, and above 80 μM inhibited cell proliferation.^67^ In general, high Zn^2+^ concentrations contribute to cell death, however, at a much lower Zn^2+^ ion concentration, Zn can even be beneficial for cell proliferation.^45^, ^62^, ^68^, ^69^, ^70^ In this work, it was presented that the amount of Zn^2+^ ions below 100.8 μM (11.4 mg/L) in pure Zn, 98.2 μM (11.1 mg/L) in the Zn‐3Ag alloy, and 118.5 μM (13.4 mg/L) in the Zn‐3Ag‐0.5Mg provide a favorable environment for cell survival and proliferation. This result is consistent with other already mentioned results for Zn‐based materials.^45^, ^62^, ^68^, ^69^, ^70^, ^71^ Furthermore, it can be seen that in the current study, the chemical composition variations of the investigated samples revealed almost no significant differences. These results are important, as it was mentioned that the use of these alloying additions is necessary to strengthen pure Zn, while simultaneously not deteriorating the biological properties of pure Zn.

### 4.3 Critical evaluation of direct contact cytotoxicity assay for Zn‐based materials

Interesting results emerged from the direct in vitro incubation of MG‐63 cells on Zn‐based samples' surfaces. The WST‐8 and LDH assays belong to the group of colorimetric assays. The principle of these assays is the biochemical reduction of the tetrazolium‐based dye included in the reagent by the NADH and NADPH, which results in a color change that can be identified with basic spectrophotometrical methods.^72^ The WST‐8 and LDH assays were used in this study to evaluate the viability of the MG‐63 cells in direct contact with metallic disks and for experiments performed on extracts. They are the most common colorimetric tests used to assess the in vitro cytotoxicity of biomaterials.^73^ However, several studies using these assays have shown misleading cell viability results. This effect was previously observed for magnesium ions in Mg‐based materials,^74^ manganese ions in Fe‐based materials,^75^ and carbon nanotubes,^76^ but it has never been raised, as an issue for biodegradable Zn alloys. Therefore, we reported for the first time the interference of Zn^2+^ ions with tetrazolium salt, the main component of the WST‐8 reagent.

For instance, it was investigated how Mn, in the form of pure particles or as an addition in Fe‐Mn powder, affects the absorbance value at particular stages of the WST‐1 assay in cytotoxicity experiments with endothelial cells. Mn is considered to interact with formazan dye, while tetrazolium salt is converted into formazan rather than with the already formed formazan dye after WST‐1 reduction. In contrast to our results, Mn ions in Fe‐Mn samples decreased the absorbance values causing an artificial loss, indicating reduced cell viability. In parallel, the test was conducted on pure Fe, showing no disturbance of the WST‐1 test.^75^ Therefore, it was suggested to use luminescence‐based assays to evaluate the in vitro cytotoxicity of Mn‐containing materials. Using MTT and XTT assays also lead to false positive or false negative results related to the cytotoxic effect of Mg‐based materials on human osteosarcoma MG‐63 cell line.^74^ It was concluded that, due to Mg's high reactivity, it participates in the enzymatic reaction of formazan formation by opening the ring form of the tetrazolium salt and binding to it, which can result in formazan color change.^74^ In the direct assay, the results are strongly influenced by actively corroding Mg samples during viability test and formazan reduction.^59^ However, it was suggested that the effect of Mg^2+^ interference should not occur in the indirect extract method since there is no actively corroding Mg samples during incubation with tetrazolium‐based reagent. Thus, these viability assays may be used for indirect measurements since the amount of ions is much lower and the enzymatic reaction is less affected by Mg^2+^ ions than it is by cells. However, the results of tetrazolium salt‐based assays must be supported using alternative techniques to prove the presented observations.

Due to the inconsistency between WST‐8 quantitative results and qualitative microscopic observations, our findings suggest recommending at least two independent test methods when verifying the cytotoxicity effect of new Zn‐based materials. In this case, the LDH assay was additionally employed to validate cell viability results. The results presented in Figure 3C indicate that, most likely, products of the anodic reaction occurring for Zn in the aqueous CCM environment, which are Zn^2+^ ions and electrons,^77^ disturb the WST‐8 assay, similarly to Mg‐based materials.^74^ The absorbance value measured for the Zn‐based samples in the test performed on metallic disks without cells indicates that the interference with ions takes place during the enzymatic reaction when formazan is formed. However, it should be pointed out that in contrast to the optical microscopic observations of the death of almost all cells surrounding of the Zn‐based disks, the absorbance value measured during tests with cells was still higher than in the test without cells, suggesting some other reason for these results. Undoubtedly, further research is required to fully characterize Zn^2+^'s interference with the tetrazolium salt‐based assay and understand the limitations of using the tetrazolium‐based assays. To explain the mechanism of Zn^2+^ ions affecting cell viability results, a series of alternative experiments should be considered for further research.

**FIGURE 10:**
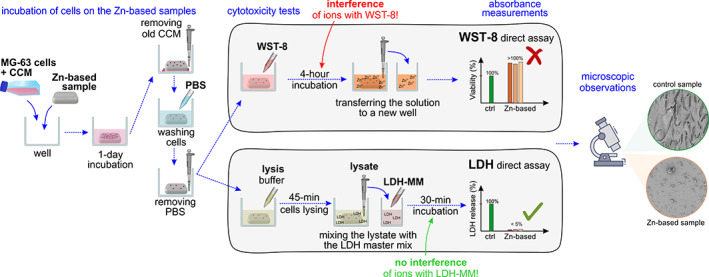
Schematic representation of the differences between the implemented protocols for WST‐8 and LDH colorimetric cytotoxicity assays. LDH, lactate dehydrogenase; WST, water‐soluble tetrazolium

Knowing that the conversion of tetrazolium salt to formazan in MTT, MTS, XTT, and WST colorimetric assays may be affected by the metallic ions,^59^, ^74^, ^78^, ^79^ dye exclusion assays, fluorometric assays, luminometric assays, and flow cytometric assays may be taken under consideration. Cytotoxicity tests, such as BrdU, LDH, or luminescence‐based, have been proposed in other studies, as they seem to be more convenient for assessing the cytocompatibility of Zn‐based materials in the direct cell culture method. Although the LDH assay belongs to the colorimetric test group and the principle is also based on the tetrazolium salt to formazan conversion, we have reported that results obtained using LDH assays are not disrupted as is the case during direct cytotoxicity test, as they indicate low LDH release levels and thus poor cell viability. It is consistent with indirect experiments, where the LDH release in 100% extract is also extremely low, proving that high Zn concentrations lead to cell death. The reason for achieving correct results in LDH assays and false–positive in WST‐8 arises from the consecutive steps in the implemented cytotoxicity test protocols. According to Figure 10, the difference lies in the point, at which the tetrazolium‐based reagent is added to the medium and if it is incubated with a metallic disk before the spectrophotometric measurements. In both assays, after 1‐day incubation of cells on the samples' surface, the old CCM is removed from the wells, and the samples with cells are carefully washed with PBS. In WST‐8, the reagent is added to the well containing metallic disks and cells. Thus, during the additional 4‐h incubation with WST‐8, Zn^2+^ ions are constantly released from the metallic sample. In theory, at this point the cells reduce WST‐8 to formazan. However, the interference of released ions with tetrazolium salt also takes place here, and as a result, formazan is formed. It leads to a change in solution color and gives overestimated cell viability results in the spectrophotometric method. In the LDH assay, the next step assumes adding a lysis buffer to the well with the metallic disk and cells, followed by short incubation periods. Next, the cell lysate is mixed in a new well with a tetrazolium salt‐based LDH reagent. It means that the tetrazolium salt included in the LDH reagent reacts only with cells and has no contact with the Zn sample and, in turn, with Zn^2+^ ions. After a short 30‐min incubation period, there is no disruption of cell viability measured spectrophotometrically.

Alternative to the tetrazolium‐based assays, other options for measuring cell viability is Alamar Blue or cell Titer Blue assays classified both as fluorometric and colorimetric tests. They contain oxidized non‐fluorescent blue resazurin that undergoes reduction to a red fluorescent resorufin dye by the mitochondrial respiratory chain in live cells. The fluorescent effect appears when the nonfluorescent compound (resazurin) is cleaved by cellular esterases. Both the fluorescent signal and color change can be measured to determine the amount of viable cells. However, greater sensitivity is achieved using the fluorescent effect.^80^, ^81^ To the best of our knowledge, there are no results reporting the interference of resazurin‐based assays with metallic ions, for example, when tested for ZnO nanoparticles.^82^ However, this assay has barely been used as a quantitative method for biodegradable metals' cytotoxicity evaluation. Since both tetrazolium‐ and resazurin‐based assays depend on enzymatic transformation, the tested samples/compounds may interfere with this reaction and lead to a false result. Therefore, using the Alamar Blue assay for cytotoxic evaluation of Zn‐based materials requires further systematic studies to eliminate a potential distortion of results.^83^

As suggested in this work, a careful approach to results interpretation should involve microscopic observations for qualitative evaluation of quantitative results obtained from colorimetric metabolic assays. Thus, it is recommended to perform a live/dead staining procedure or a cell fixation procedure for SEM investigations to validate the results.

### 4.4 Antibacterial activity—beneficial property in implant materials

In addition to biocompatibility, antibacterial activity is another critical property for biodegradable implants, which could help prevent biofilm formation and infections. It can be a valuable characteristic of the biodegradable material since it would diminish the inflammation risk and the possibility of immunological rejection. It was reported that the incidence rate of surgical site infection, accounting globally for about 10%–20% of all nosocomial infections, represents the most common type of health care‐associated infection. The most frequently reported microorganisms are Gram‐negative *E. coli* and Gram‐positive *S. aureus* causing implantation site infection in surgical procedures.^84^

Typically, the additions of ions, such as Ag and Cu, to metallic alloys inhibit bacterial growth.^61^ Up until now, research has confirmed an antibacterial activity of Ag‐rich or Cu‐rich Zn‐alloys in vitro.^23^, ^36^, ^85^, ^86^ Generally, the possible key factors responsible for the antibacterial activity of biodegradable metallic materials can be attributed to changes in pH during their degradation and the direct impact of released free metallic ions on bacteria.^87^ With the corrosion of Zn or Mg, the increasing amount of OH^−^ ions contributes to the growing alkalinity of the solution and thus decreases bacteria viability, as it is believed that most bacteria can only survive in a pH range of 6–8.^88^ However, Zn itself demonstrates antibacterial properties against different pathogens.^89^ According to the Pourbaix diagram, in an environment of pH <7.4, pure Zn tends to release free Zn^2+^ ions,^90^ directly impacting bacteria viability. The potential mechanism of bacterial growth reduction can be related to (1) direct electrostatic interactions between the positively charged Zn^2+^ ions with the negatively charged bacterial membranes, leading to impaired membrane function and nutrient assimilation; (2) formation of extracellular and intracellular reactive oxygen species (ROS), and damage of lipids, proteins, and DNA by oxidative stress; (3) damage to the plasma membrane and thus leakage of the cell content due to high‐ROS levels and metal binding to the cell envelope; (4) interference of ions with both proteins and DNA, impairing their function, disturbing cellular metabolism and finally resulting in bacterial death.^87^, ^91^, ^92^

The Gram‐positive bacteria are reported to be more susceptible to Zn^2+^ ions than Gram‐negative bacteria, which has been associated with the difference in the protein constituents of their cell walls.^35^ Although we have not observed drastic variations in antibacterial behavior of Zn‐based samples for the studied bacteria, the effect on *S. aureus*, that is a Gram‐positive bacterium, is slightly more noticeable considering the size of the bacterial growth inhibition zone and the relative bacteria viability in suspensions.

Ag is a beneficial element for imparting antibacterial properties to implants. The antibacterial effect of Ag is well‐known and repeatedly demonstrated when added to various materials used for biomedical applications. The antibacterial mechanism is related to morphological and physiological changes of bacterial cells exposed to Ag^+,^ causing inhibition of their replication ability.^35^ It was shown that Ag^+^ ions inhibit the propagation of microorganisms such as bacteria, yeasts, viruses, and fungi.^93^ However, in the current studies, the effect of Ag addition on the antibacterial activity is not so evident, especially in turbidity tests. Zn^2+^ ions seem to play a much more significant role in inhibiting bacterial growth. Antibacterial activity results showed that the Zn‐3Ag‐0.5Mg alloy inhibited 50% of *S. aureus* over 6 h and of *E. coli* within 24 h. Although these findings do not support complete bacterial inhibition by any of the alloys against both bacteria types, the Zn‐3Ag‐0.5Mg alloy is the most promising material, as, in vitro, it exhibits the ability of dissociated ions to reduce the viability of *S. aureus* and *E. coli*. Therefore, the effect of the analyzed metallic ions on antibacterial properties is ambiguous as previous research using Zn with Ag and Mg additions concluded that these ions possess much greater antibacterial properties against common bacteria, including *S. aureus* and *E. coli*.^28^, ^61^, ^94^, ^95^

A smaller inhibition zone diameter against both bacterial strains formed around the Zn‐3Ag‐0.5Mg alloys. It probably results from the lower corrosion rate and thus a lower amount of released Zn^2+^ ions than the other samples. Interestingly, contrary to the agar well diffusion assay, the indirect colorimetric assay does not significantly differ between the alloys. This phenomenon could be due to the ion release rate of Zn‐3Ag‐0.5Mg alloys in the aqueous media rather than incubated in agar in fixated positions. In this case, this alloy may have released Zn, Ag, and Mg ions at a high rate, resulting in reduced relative bacterial viability. This result is consistent with ICP results, as depicted in Figure 6D.

Our study has mainly focused on in vitro evaluation of cytotoxicity and antibacterial activity of promising the Zn‐3Ag‐0.5Mg alloy in terms of its high potential application as a biodegradable implant material. Based on the presented results, a large concentration of Zn2+ ions released from implants should be carefully controlled to avoid cytotoxicity, provide a suitable environment for cell growth and proliferation, and simultaneously inhibit bacterial growth. It should be highlighted that this study is the first to demonstrate limitations related to water‐soluble tetrazolium‐based direct cytotoxicity assays. Therefore, a more suitable cytotoxicity assay should be carefully chosen for Zn‐based materials as they may interfere with tetrazolium salt, leading to formazan conversion and false‐positive results.

## 5 CONCLUSIONS

In this work, Ag and Mg alloying additions to the in vitro cytotoxicity and antibacterial properties of Zn‐3Ag and Zn‐3Ag‐0.5Mg alloys were investigated. The main conclusions that can be drawn are as follows:

1. Cytotoxicity tests showed almost no significant differences between pure Zn and Zn alloys. Therefore, changes in the chemical and phase composition invoked in the fabricated Zn alloys by alloying with small Ag and Mg additions do not affect cell viability. In the direct contact test, the cytotoxicity on MG‐63 cells after 1‐day incubation was possibly induced by high Zn^2+^ ion release to the cell culture medium.
2. The indirect extract‐based test showed a dose‐dependent cytotoxic effect on MG‐63 cells by Zn^2+^ ions. The diluted extracts of Zn, Zn‐3Ag, and Zn‐3Ag‐0.5Mg alloys showed no cytotoxicity toward MG‐63 cells at a concentration of ≤12.5%. The cytotoxic effect was observed only at high Zn^2+^ ion concentrations. The LD_50_ value for MG‐63 cells was determined as follows: 11.4 mg/L for Zn, 11.1 mg/L for the Zn‐3Ag alloy, 13.4 mg/L for the Zn‐3Ag‐0.5Mg of Zn^2+^ ions.
3. The indirect colorimetric assay indicates that all investigated materials exhibit similar antibacterial activity against *E. coli* and *S. aureus* bacteria. The Zn‐3Ag‐0.5Mg alloy inhibited 50% of *S. aureus* over 6 h and of *E. coli* within 24 h. The Agar well diffusion assay showed bacteria growth inhibition zone formation around all Zn‐based tested samples. However, the Zn‐3Ag‐0.5Mg alloy had the lowest inhibition zone diameter, which is in agreement with the ICP results.
4. Cell viability evaluation based on the direct contact test gives misleading, false‐positive results when the cytotoxicity is measured with the WST‐8 assay. The limitation of using a water‐soluble tetrazolium salt‐based assay for cytotoxicity assessment derives from the interference of Zn^2+^ ions (released from the metallic disk immersed in CCM) with tetrazolium salt included in the WST‐8 reagent. The performed research indicates that Zn^2+^ ions participate in tetrazolium salt to formazan conversion, causing solution color change measured in further steps and related to cell viability.
5. Water‐soluble tetrazolium salt‐based cytotoxicity assays, such as WST‐8, are inappropriate for direct contact tests on Zn‐based samples. The cell viability assay used for indirect extract‐based tests should be supported by other experimental techniques, for example, fluorescent microscopic observations of properly stained cells allowing for the validation of the quantitative results or validated with other alternative cytotoxicity assays, such as LDH, which do not interfere with Zn^2+^ ions or Alamar Blue (resazurin‐based) to provide reliable cell viability results.

The presented results indicate that Ag and Mg additions necessary for strength and ductility enhancement do not significantly alter Z's biological behavior, including biocompatibility or antibacterial properties. Therefore, considering our previous mechanical studies^15^, ^16^ and satisfactory biodegradation behavior,^17^ the Zn‐3Ag‐0.5Mg alloy exhibits promising properties for potential application as a biodegradable implant material.

## CONFLICT OF INTEREST

The authors declare no potential conflict of interest.

## Data Availability

The authors confirm that all relevant data are shown within the manuscript.
